# Composite acoustic system for low-frequency sound absorption

**DOI:** 10.1038/s41598-025-89151-5

**Published:** 2025-02-08

**Authors:** Eulalia Gliścińska, Marina Michalak

**Affiliations:** Faculty of Material Technologies and Textile Design, Lodz University of Technology, Textile Institute, 116 Zeromskiego Street, Lodz, 90-924 Poland

**Keywords:** Composite, Polymer surface layer, Low frequency, Sound absorption, Distance, Angle, Engineering, Materials science

## Abstract

Absorption of low-frequency sound is a difficult engineering problem because long-wave sound energy is poorly dissipated. Traditional sound absorbing materials such as porous materials, microperforated sheets or sound absorbing wedges have a poor low-frequency sound absorption performance. In this work a porous composite absorbing plate connected with a resonant cavity is developed to absorb low frequency sounds. The special developed sound-absorbing plate is rigid and consists of a more fibrous layer (up to approx. 3.0 mm thick) and a more plastic, thin polymer surface layer (up to approx. 0.5 mm thick). The walls of resonant cavity are rigid and smooth. By changing the angle between the absorbing plate and the direction of the incident sound wave and changing the length of the cavity, it is possible to create acoustic systems with a given level of sound absorption and in a given low-frequency range. The larger the angle, the maximum absorption occurs for sounds of lower frequencies. As the cavity length increases, the range of maximum absorption occurring at the resonant frequency shifts towards lower frequencies and the maximum value of the sound absorption coefficient increases. The results are compared with composite variant without a polymer surface layer.

## Introduction

Caring for the natural environment is one of the priorities of the present world. Man is exposed to various types of pollution negatively affecting the quality of life and health. One of the factors of environmental pollution is noise, especially the low-frequency noise, which can cause e.g. hearing loss, headaches, sleep disturbances, inattention, discomfort. According to the World Health Organization (WHO), one in five Europeans is regularly exposed to noise levels at night that can significantly impair health.

Sound is an energy wave that travels in all directions through air, water and solids. The number of waves per second is measured in hertz (Hz). The shorter the wavelength, the higher the frequency of the sound.

Low frequency sounds can be perceived as vibrations and affect human health. Especially the sounds at the 20 Hz border can be particularly acute.

Unfortunately, it is not possible to reduce all vibrations simultaneously and to the same low level. A very important line of research for engineers is the development of sound-absorbing materials designed to absorb sounds in a given frequency range, but also intended for a given type of application.

Absorption of low-frequency sound is a difficult engineering problem because long-wave sound energy is poorly dissipated. Traditional sound absorbing materials such as porous materials, microperforated sheets or sound absorbing wedges have poor low-frequency sound absorption performance. To improve this, porous graded materials and multilayer structures are developed or the microstructure of the material is optimized^1^. The literature on the subject includes many publications on low-frequency sound-absorbing materials, from the use of microperforated panels, also connected to a void^2^ or porous metal and voids^3^, ultra-thin panels^4^, porous materials^5,6^, Helmholtz resonators or metamaterials^7–13^.

In order to improve sound absorption in the low-frequency area, microperforated plate absorbers supported by Helmholtz resonators^14^, thin microperforated plates with holes penetrated by copper fibers, which can extend the absorption band and improve low-frequency sound absorption^15,16^, parallel microperforated plates with an air layer between them and without any rigid banking^17^, combined structures such as fiber layers with an outer layer of a perforated composite^18^, composite hybrid porous metamaterials with broadband sound absorption^19–21^, are proposed.

Sound-absorbing products are made of various materials, with a different structure, thickness, surface type and stiffness. Usually, sound absorbers are made of metal, foam, or composite, e.g. based on wood, rubber^22,23^, rice-husk^24^, rice straw^25^, natural fibers, recycled fibers^26–28^.

Fiber-based materials such as nonwovens are being developed more and more. These are porous materials, often used as systems of layers, differing in structure resulting from technology, technological conditions, and the fibers used. The type of fibers, their diameter and packing density are the main factors determining the degree of sound absorption. However, nonwovens best absorb mid- and high-frequency sounds^29^. It is troublesome to ensure absorption of low-frequency sounds. In the case of composites based on nonwovens, the ability to absorb sounds of different frequencies can be influenced to a greater extent. It is possible to produce a composite with a different layered structure, combine a perforated panel with a porous structure, use voids between the composite layers, and profile the composite surfaces. Adding air spaces behind the tested composite systems could improve the sound absorption at low and mid frequencies^29–32^.

Research shows that sound absorption may depend on the type of sound field.

The theoretical estimation of sound absorption curves depends on the characteristics of the sound field, whether it is normal, or random or circular with a range of incidence angle is from 0° to 90°^33^. According to the literature^34^, from the point of view of physical considerations, maximum absorption should occur at normal incidence, which was confirmed by the test results. The sound absorption coefficient may vary with fluctuation of pressure on the absorbing material at the constant intensity of the incident sound. Moreover, the sound absorption coefficient may vary at different angles of incidence. This relationship is not consistent with Paris’s formula according to which the coefficient of 0.28 at normal incidence increases by approximately 25% at 45 degrees and by 50% at 60 degrees. According to^35^, in the high frequency range from 4 kHz to 40 kHz there is no significant dependence of the sound absorption coefficient of materials on the sound wave incidence angle. According to the literature^36^, the sound absorption coefficient in the low frequency range from 1 Hz to 1000 Hz increases with increasing incidence angle. A method for estimating the angle - sound absorption coefficient dependence for a large material sample using a compact microphone array has even been developed^37^.

According to the literature^38^, theoretical considerations for Helmholtz resonator-type structures have shown the dependence of the noise suppression ability on the geometric shapes of the resonator. Furthermore, a traditional, rigid Helmholtz resonator can only provide maximum absorption at one frequency.

The presented work concerns a porous composite absorbing plate connected with a resonant cavity with an adjustable low frequency sound absorption. Research aimed at obtaining a system that absorbs low-frequency sounds showed the possibility of adjusting the geometry of the acoustic system to the absorption of sounds of selected frequencies.

By changing the geometry of the acoustic system, as the length of the cavity, the angle between the absorbing plate and the direction of the incident sound wave, one can influence its sound absorption measured with an impedance tube. This dependence applies not only to the value of the maximum absorption coefficient, but also to the sound frequency range of the maximum absorption. The structure of the composite absorbing plate, especially its surface layer, also influences sound absorption.

The proposed acoustic system, which can be multiplied in real conditions, works similarly to a Helmholtz resonator and consists of a porous absorbing plate and a cavity. The absorbing plate is a composite material based on a thermoplastic matrix, produced from nonwovens in a pressing process. The composite material consists of two parts, i.e. a more fibrous layer (up to approx. 3.0 mm thick) and a more plastic, thin polymer surface layer (up to approx. 0.5 mm thick). Moreover, by producing a composite material only from fibers, it can be selected in a way that ensures its biodegradability, and thus it can have a positive impact on the environment^5,18^. The fibers used in the work are made of polymers obtained from renewable sources. The proposed material is environmentally friendly.

## Research material

### Acoustic system with absorbing plate and resonant cavity

**Fig. 1:**
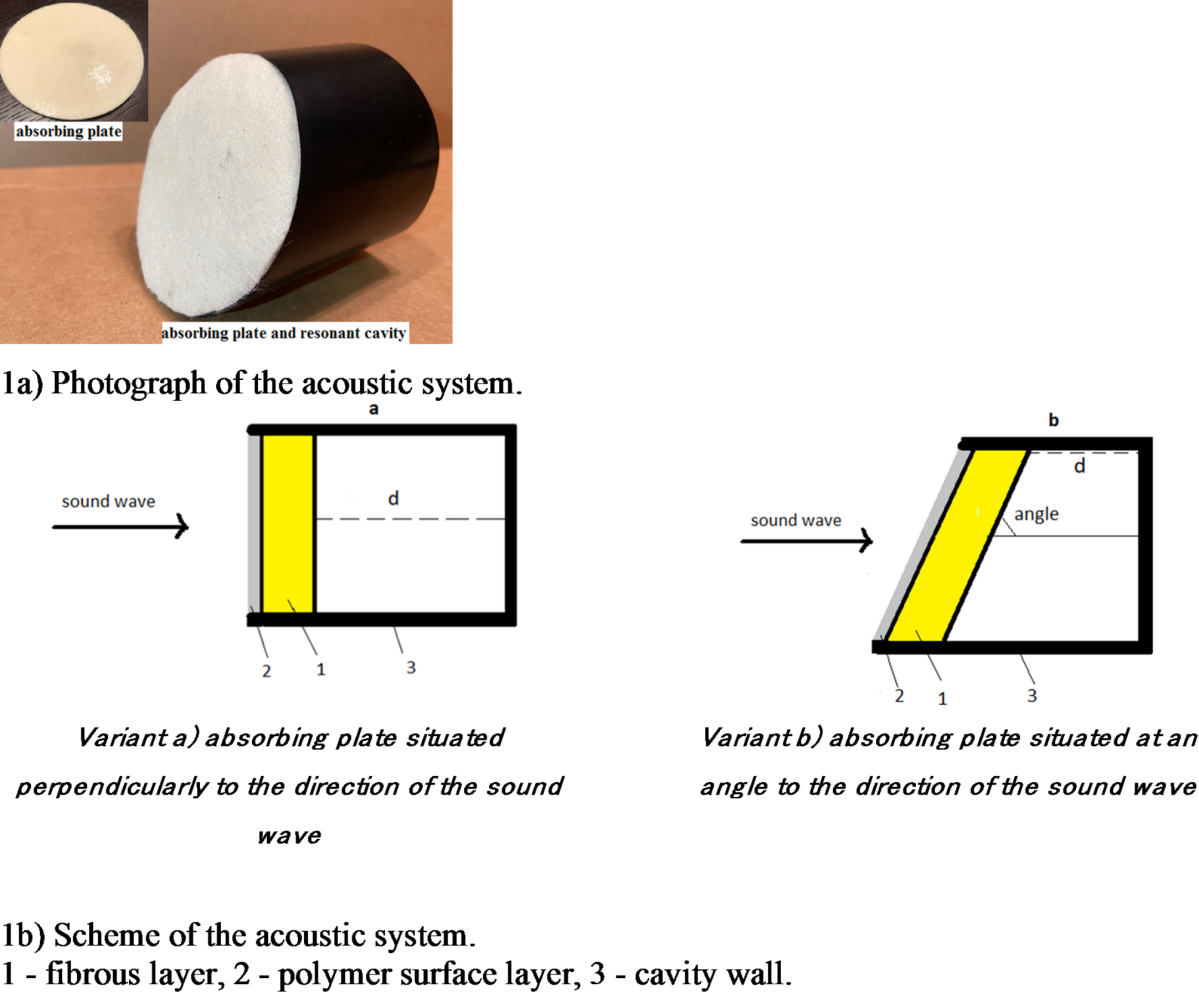
Acoustic system.

The acoustic system consisting of a porous absorbing plate and a resonant cavity was investigated, Fig. 1a. As the absorbing plate was used a composite material consisting of a more fibrous part covered on one side with a more plastic thin polymer surface layer. On the fibrous side, the absorbing plate was connected to a cavity filled with air. In the tests, the absorbing plate had the polymer side facing the sound wave. The dependence of the system sound absorption coefficient on the cavity length and the orientation angle of the absorbing plate in relation to the direction of the incident sound wave was investigated. Two variants of the acoustic system were used in the research, differing in the geometry of the cavity:

***Variant a*** - absorbing plate situated perpendicularly to the direction of the sound wave, connected to the cylinder cavity at the back, Fig. 1b Variant a. The length of the cavity (distance “d”) between the absorbing plate and the opposite back wall varied up to 100 mm with an accuracy of 1 mm.

***Variant b*** - absorbing plate situated at an angle to the direction of the sound wave, connected to the cylinder cavity at the back, Fig. 1b Variant b. The angle between the absorbing plate and the direction of the incident sound wave in the acoustic system had five different values: arctg2 (63°), arctg2.5 (68°), arctg10/3 (73°), arctg5 (79°), arctg10 (84°), and the shortest distance “d” between the absorbing plate and the opposite back wall up to 50 mm with an accuracy of 1 mm.

The cavity walls consisted of an impedance tube. The cavity diameter was 100 mm in all cases, on the length “d”.

### Absorbing plate

The absorbing plate was cut from composite material. Textile products, i.e. needle-punched nonwovens, were used to produce the composite material. This material was obtained by pressing a system of nonwoven layers, i.e. layers of nonwoven fabric (PLA/Vi) made of thermoplastic fibers and reinforcing fibers and layers of nonwoven fabric (PLA) made of thermoplastic fibers, placed on top of the system.

Each composite material was produced in the form of a hydraulic press with a water cooling system from Hydromega Sp. z o. o., Poland. The pressing process consisted of three stages:

- heating - up to 170 °C, time: 15 min, without pressure,
- consolidation - temperature: 170 °C, time: 5 min, pressure: 0.27 MPa,
- cooling – to room temperature, time: 15 min, pressure: 0.27 MPa.

After the pressing process, the mixed nonwoven fabric (PLA/Vi) layers formed the more fibrous and thicker part of the composite material, and the thermoplastic nonwoven fabric (PLA) layers formed a more plastic and thinner polymer layer.

Polylactide fibers (PLA) 6.7 dtex / 64 mm, under the name of Ingeo Fiber SLN2660D, with a finishing composition containing polylactide resin and no hazardous compounds, supplied by the Far Eastern Textile Ltd. (Taiwan) were used to obtain the thermoplastic matrix of the composite material. The melting point of PLA fibers is in the temperature range of 165–170 °C. As a reinforcement of the composite material the man-made viscose (Vi) fibers with linear density of 4.4 dtex and length of 70 mm were used.

The characteristics of five variants of layer systems numbered from 1 to 5 and the composite materials obtained from them are presented in Table 1. The thickness of the composite materials was measured using a micrometric screw. In order to measure the thickness of the polymer surface layer, it was mechanically separated from the fibrous part. For comparison, a reference sample without polymer surface layer was also produced, labeled “0”.

**Table 1:** Characteristics of the composite materials.

No. of composite material/no. of thickness of polymer surface layer	System of nonwoven layers before pressing	Composite structure	Total thickness, mm	Thickness of polymer surface layer, mm
0	8 layers (PLA/Vi)	Fibrous	2.05 ± 0.01	-
1	7 layers (PLA/Vi) + 1 layer (PLA)	Fibrous/plastic	2.87 ± 0.01	0.26 ± 0.01
2	7 layers (PLA/Vi) + 2 layers (PLA)	Fibrous/plastic	2.72 ± 0.01	0.34 ± 0.01
3	7 layers (PLA/Vi) + 3 layers (PLA)	Fibrous/plastic	2.93 ± 0.01	0.35 ± 0.01
4	7 layers (PLA/Vi) + 4 layers (PLA)	Fibrous/plastic	2.85 ± 0.01	0.40 ± 0.01
5	7 layers (PLA/Vi) + 5 layers (PLA)	Fibrous/plastic	2.74 ± 0.01	0.52 ± 0.01

The sound absorption tests were carried out for acoustic systems containing absorbing plates cut from the individual composite materials mentioned. Due to the large number and repeatability of the results and in order to make the paper more transparent, the article is limited to presenting the results of acoustic systems with absorbing plates cut from selected composite materials.

## Research methodology

**Fig. 2:**
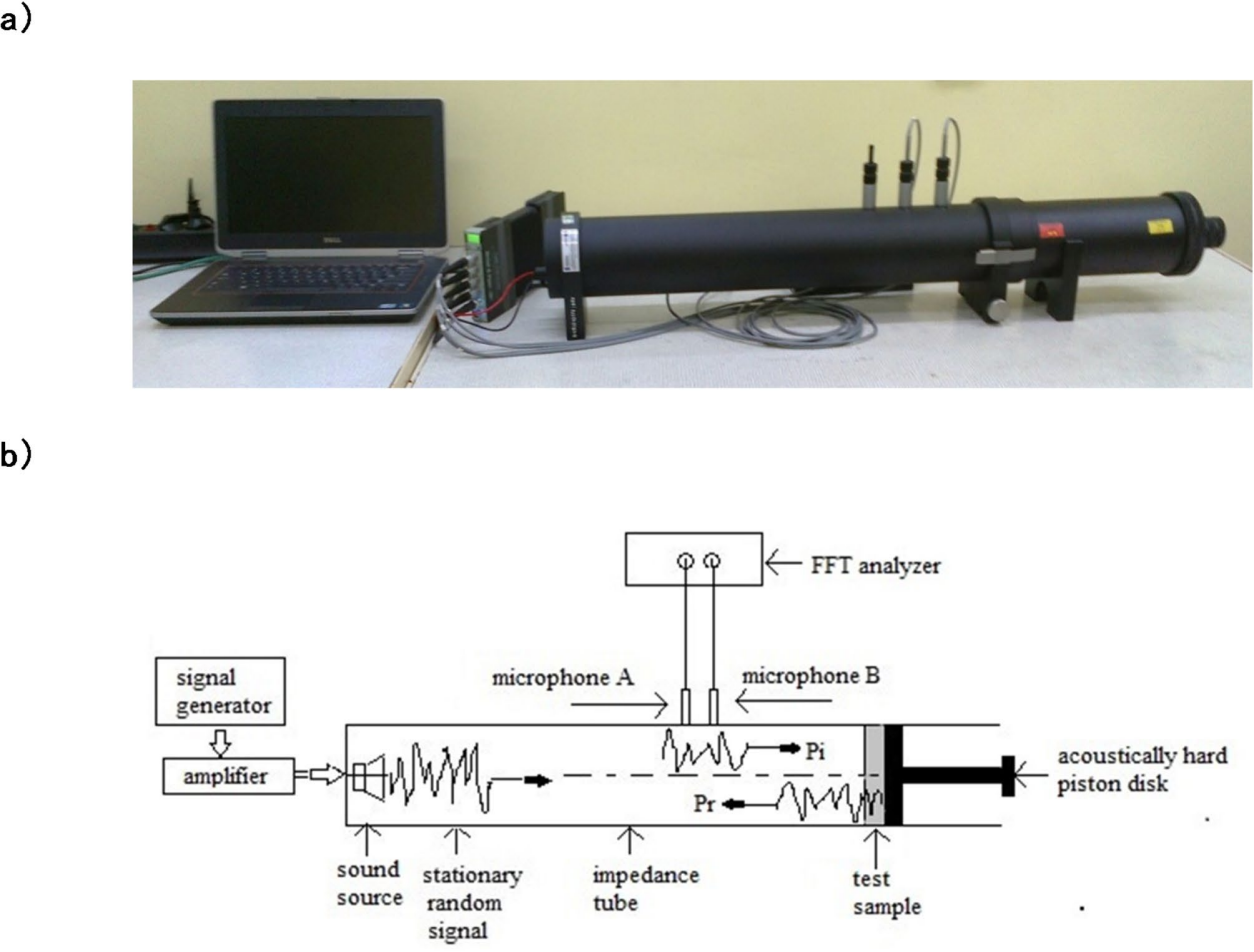
Research device for testing acoustic properties (Brüel & Kjaer, Denmark); (**a**) real device, (**b**) device diagram; FFT - two-channel fast Fourier transform analysis system.

Sound absorption of composite systems was determined based on the sound absorption coefficient. An impedance tube, the so-called Kundt tube, type 4206, with a diameter of 100 mm and a low-frequency range of normal incidence wave (from 50 Hz to 1600 Hz) was used, Fig. 2a. The sound absorption coefficient was determined by the two-microphone technique in accordance with the ISO 10534-2 standard^39^, Fig. 2b.

The two-microphone technique of measuring the sound absorption coefficient is based on the assumption that, for a perpendicularly incident sound wave, the reflection coefficient can be determined from the measured transfer function H_12_ between two single microphones placed on the wall of the tube. The technique uses the decomposition of a broadband stationary random signal produced by a sound source into its incident (Pi) and reflected (Pr) components. These components are determined from the relationship between the sound pressure measured by microphones at two positions on the tube wall. Three functions are calculated from the incident and reflected sound pressure components at the two microphone positions:

H_12_ - transfer function between microphones 1 and 2,

H_i_ – transfer function for the incident wave,

H_r_ – transfer function for the reflected wave.

Using the transfer function method the sound reflection coefficient is calculated:

1 $$\:\text{r}=\left(\frac{\text{H}12\:-\:\text{H}\text{i}}{\text{H}\text{r}\:\--\:\text{H}12}\right)\: {\rm e} ^{\rm 2jkx}$$

where:

k – wave number,

x – the sample distance from the farther microphone.

The sound absorption coefficient is calculated from the following equation:

2 $$ \alpha \:= 1 - |r| ^{2} = 1 - | \:\left(\frac{\text{H}12-\:\text{H}\text{i}}{\text{H}\text{r}\:\--\:\text{H}12}\right)\: {\rm e} ^{\rm 2jkx }| ^{2}.$$

## Theoretical part

**Fig. 3:**
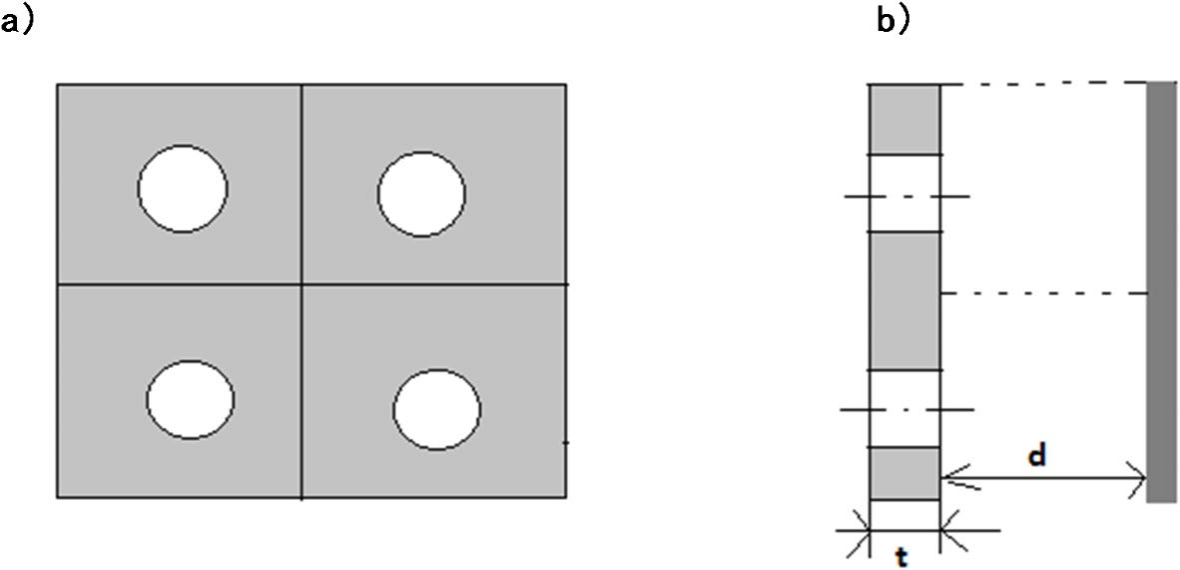
Resonant sound absorber with perforated panel by S.N. Rzhevkin, on the basis of^40^; (a) front view, (b) side view; t – composite thickness, d – cavity length.

In the literature^40^ the simplest resonant sound absorber is a perforated panel with holes, placed at a certain distance from a rigid wall. This type of absorber was first proposed by the Soviet acoustician S.N. Rzhevkin in 1938^41,42^. Scheme of this resonant sound absorber is shown in Fig. 3. In our presented work the acoustic system with an absorbing plate and resonant cavity is investigated. Due to its fibrous nature, the absorbing plate made of textile products is characterized by specific porosity, therefore it can be presented as a perforated panel with holes as in Fig. 3a. The distance between the inner surface of the absorbing plate and opposite back wall is a cavity length, i.e. the distance from the inner surface of the panel to the rigid wall as in Fig. 3b.

The considered structure is an oscillatory system in which the elastic element is the air filling the space behind the panel, and the inertial element is the air plugs filling the panel holes. As the frequency of the sound wave incident on the front surface of the sound absorber approaches the natural frequency of the resonator, the speed of air movement in the panel holes increases dramatically. At the same time, the loss of acoustic energy due to viscous friction forces increases^40^.

The acoustic properties of the material with the cavity are characterized by the input impedance Z and the sound absorption coefficient ∝^43,44^. Theoretical assumptions contained in the mentioned literature have been applied to the acoustic system presented in this article.

The input impedance of the sound absorbing material is, by definition, equal to the ratio of the sound pressure $\:p$ to the normal component of the oscillation velocity $\:{v}_{n}$, i.e. the number of movements (oscillations) per unit time, and in the general case is a complex quantity:

3 $$\:Z=\frac{p}{{v}_{n}}=R+iY$$

where:

*R* - active acoustic resistance - determined on the basis of energy losses in the acoustic system,

$\:iY$*-* imaginary part - reactive part, resulting from the elasticity or inertia of the mass.

Typically, the so-called dimensionless or normalized impedance $\:{Z}_{1}$ is used:

4 $$\:{Z}_{1}=\frac{Z}{{\rho\:}_{o}c}={R}_{1}+i{Y}_{1}$$

where:

$\:{\rho\:}_{o}c$ - wave resistance of the environment.

For air under normal conditions it is:

$$\:{\rho\:}_{o}=\text{1,27}\:\frac{kg}{{m}^{3}},\:c=343\:\frac{m}{s},$$

The sound absorption coefficient is defined as the inverse of the distance over which the amplitude of a sound wave decreases e times.

The imaginary (reactive) component of the impedance Y_1_ is determined by the elasticity of the air in the volume of the resonant cavity and the inertia of the air oscillating in the vicinity of the holes of the perforated panel:

5 $$\:{Y}_{1}=\frac{\omega\:\left(t+2\delta\:\right)}{\eta\:c}-ctg\frac{\omega\:d}{c}$$

where:

$\:d$ – cavity length - depth of the resonant cavity (the distance from the inner surface of the panel to the rigid wall),

$\:\eta\:\:$*-* front surface perforation ratio is equal to the ratio of the hole area to the square cell area per hole,

$$\:t$$ – composite thickness,

$\:2\delta\:$ – correction related to diffraction phenomena.

At resonance ($\:f={f}_{res}$) Y_1_ = 0 and the sound absorption coefficient of the system α reaches its maximum value.

To determine the resonant frequency f_res_, the equation should be solved:

6 $$\:\frac{2\pi\:{f}_{res}\left(t+2\delta\:\right)}{\eta\:c}=ctg\frac{2\pi\:fd}{c}$$

In the general case, this equation requires a numerical solution, however, provided that the sound wavelength $\:\lambda\:$ >> l (or 2$\:\pi\:fd/c$ << 1), we can consider

7 $$\:ctg\frac{2\pi\:fd}{c}\approx\:\frac{c}{\begin{array}{c}2\pi\:fd\\\:\end{array}}$$

Leaving out the long leads, the final result is:

8 $$\:{f}_{res}\approx\:\frac{{\rm c}}{2\pi\:}\sqrt{\frac{\eta\:}{\left(t+2\delta\:\right)d}}$$

$$\:{f}_{res}$$ – resonant frequency,

$\:2\delta\:$ is much smaller than t.

Since it is not possible to precisely calculate the dimensions of the holes in the composite, we will use a simplification:

9 $$\:{f}_{res}\approx\:\frac{{\rm c}\sqrt{\eta\:}}{2\pi\:}\sqrt{\frac{1}{t\cdot\:d}}\:=\:\text{Q}\sqrt{\frac{1}{d}}$$

where:

10 $$\:Q=\frac{{\rm {c}}}{2\pi\:}\sqrt{\frac{\eta\:}{t}}$$

The value Q is received for one freely selected value from experiment that allows us to calculate the dependence of the resonance frequency on the dimensions of the cavity, which we will present later.

## Results and discussion

### Sound absorption of the absorbing plate - the influence of the thickness of the polymer surface layer

**Fig. 4:**
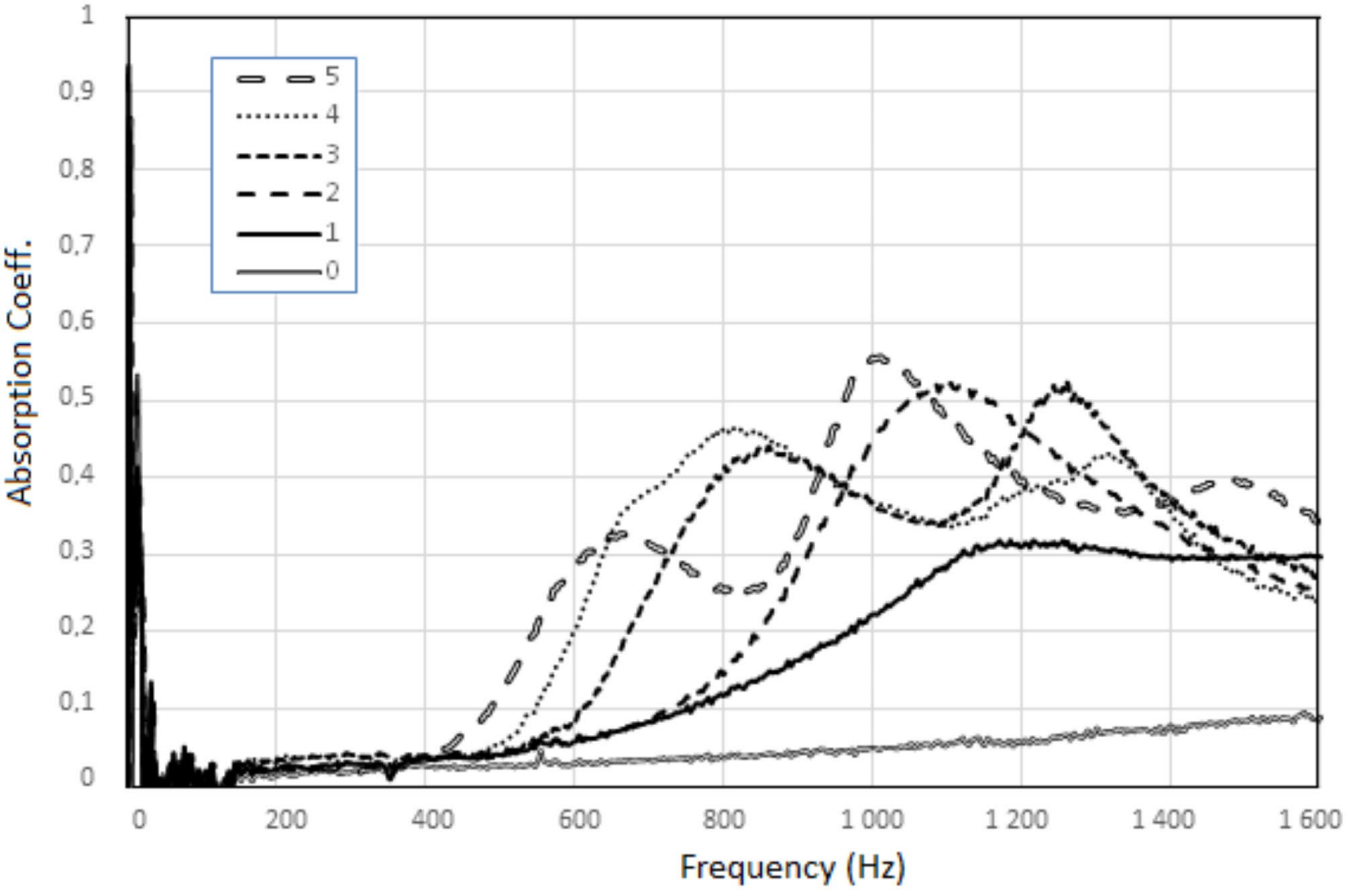
Sound absorption of the composite absorbing plate situated perpendicular to the direction of the sound wave - different thickness of the polymer surface layer (as in Table 1).

The results of the sound absorption coefficient for sound frequencies up to 1600 Hz for individual absorbing plates with a polymer surface layer are shown in Fig. 4.

The conducted works confirmed the results obtained from previous tests for similar materials, but carried out for the sound frequency range of 1500–6400 Hz^45^. It has been shown that the porous composite material absorbs sounds to a lesser extent than the same material but with an additional thin polymer surface layer situated on the side of the incident sound wave. For the above materials, the nature of the dependence of the sound absorption coefficient on the sound frequency is also differential. For the range of low frequencies, i.e. up to 1600 Hz, for a fibrous composite material, the sound absorption coefficient increases slightly with increasing frequency. On the other hand, for a fibrous composite material with a polymer surface layer, the sound absorption coefficient increases significantly with increasing frequency, and then settles at this level or lowers creating a peak or peaks indicating the frequency range of the most absorbed sounds, Fig. 4.

### Sound absorption of the acoustic system with absorbing plate and resonant cavity - the influence of the cavity length

#### Acoustic system with absorbing plate situated perpendicularly to the direction of the sound wave - variant a

The dependence of the sound absorption coefficient of the system on the cavity length was investigated. The tests used a cavity in the form of a cylinder, the front of which was an absorbing plate situated perpendicular to the direction of the sound wave. The distance “d” between the absorbing plate and the opposite back rigid wall of the cavity was up to 70 mm, Fig. 1b Variant a.

**Fig. 5:**
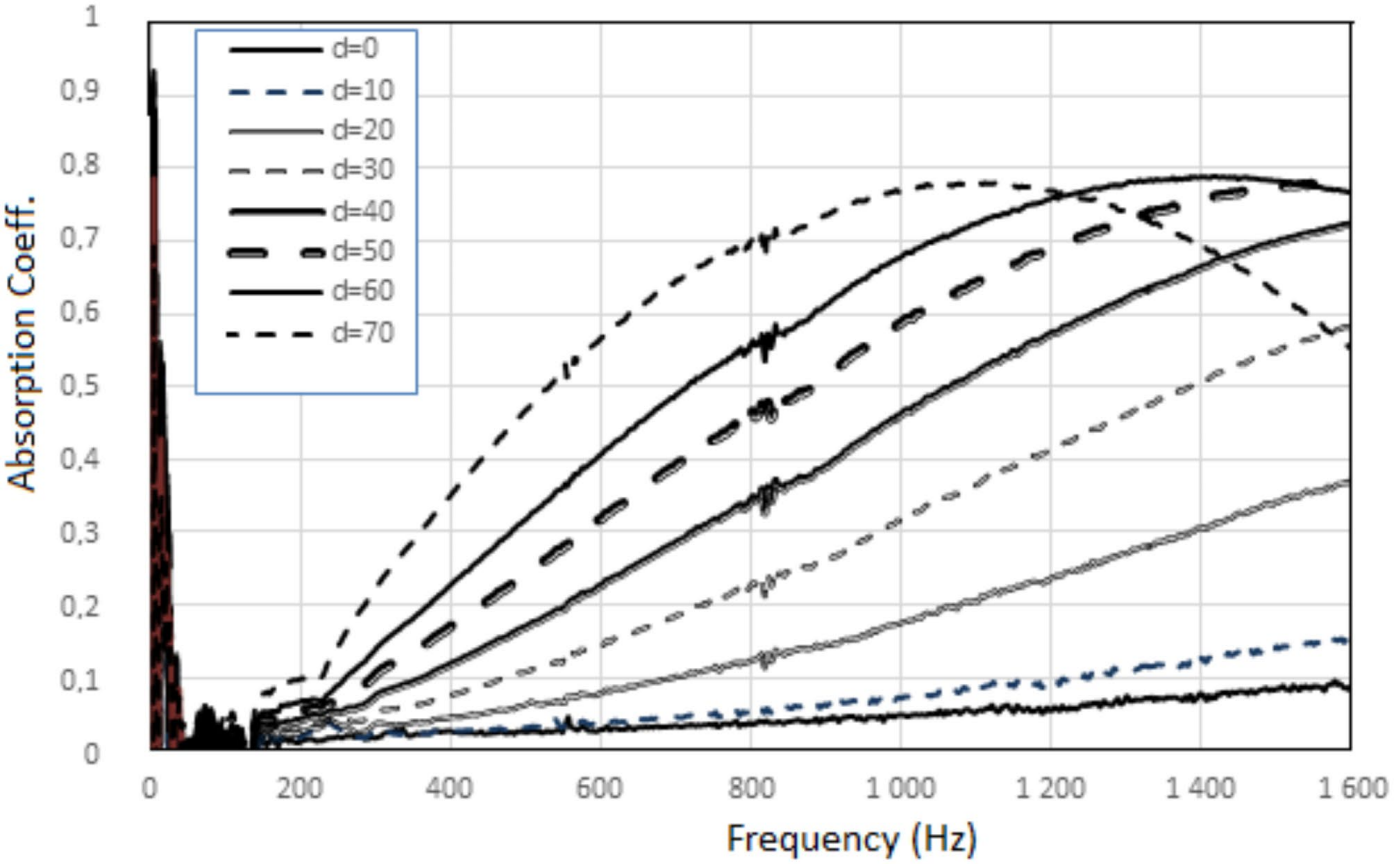
Sound absorption of the acoustic system with a reference absorbing plate (without a polymer surface layer) situated perpendicularly to the direction of the sound wave - different cavity length.

**Fig. 6:**
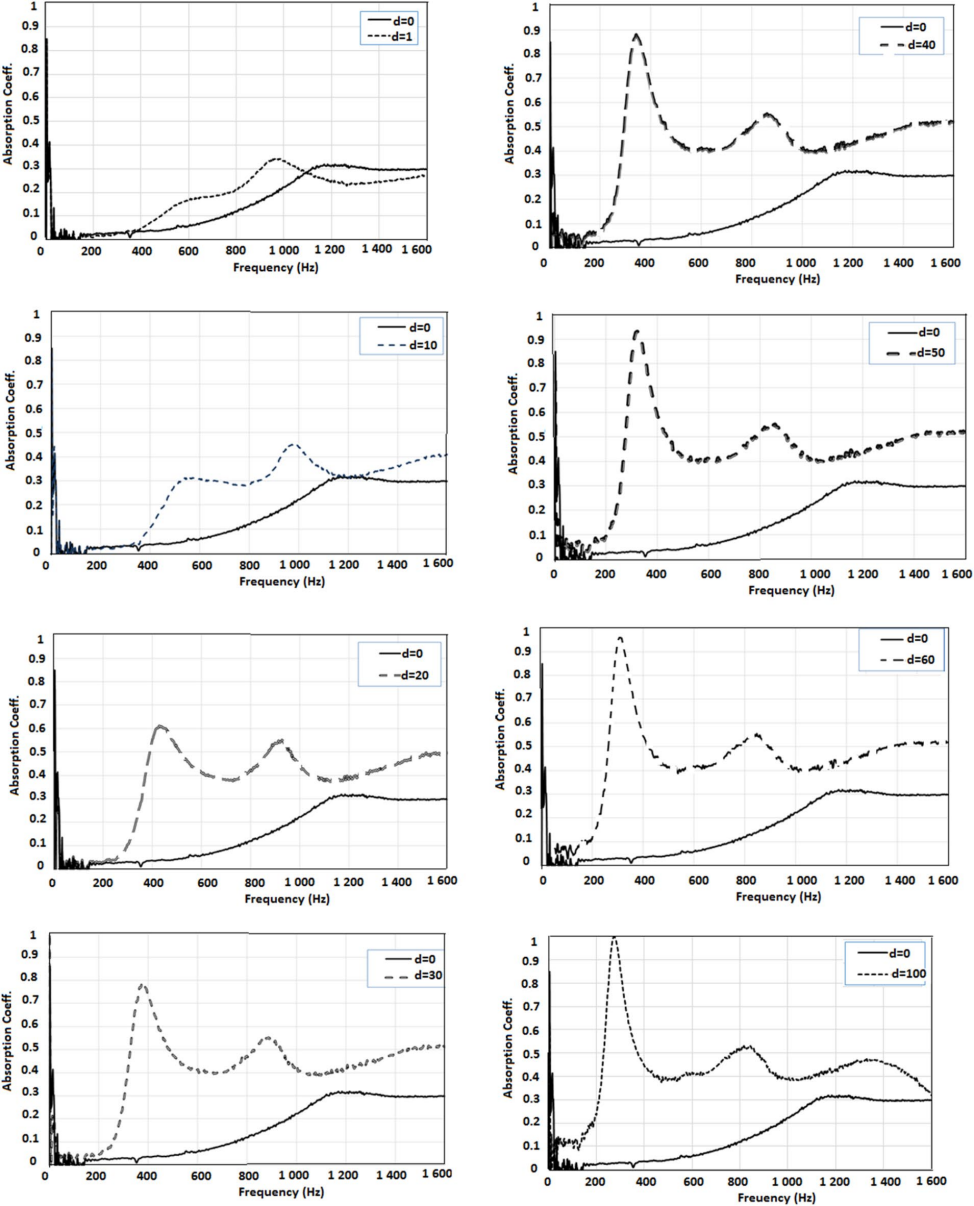
Sound absorption of the acoustic system with an absorbing plate containing a polymer surface layer, situated perpendicularly to the direction of the sound wave - different cavity length (separate drawings for subsequent cavity length “d”).

The results of the sound absorption test as a function of the sound frequency wave for a different cavity length and without cavity (d = 0) are shown in Figs. 5 and 6.

A system with an absorbing plate without a polymer surface layer, Fig. 5, has no visible resonant sound frequency. Generally, the longer cavity length ‘d’, the greater sound absorption over the entire frequency range, up to the optimal value of “d”, after which the nature of the curve changes.

A system with an absorbing plate containing a polymer surface layer has visible resonant sound frequency, Fig. 6.

Only the peak dependent on the length “d” is analyzed, while other peaks arising at higher frequencies due to the properties of the fibrous part of the composite, are not analyzed.

In the case of a system with a sound-absorbing plate containing a polymer surface layer, for all tested cavity lengths, the maximum sound absorption is higher and occurs at lower frequencies, Fig. 6, than in the case of a system with a sound-absorbing plate without a polymer surface layer, Fig. 5.

From Fig. 6 it can be seen that in the system without a cavity (distance “d” equal to 0), sound absorption coefficient greater than 0.1 is observed from 800 Hz to the end of the tested frequency range. The highest sound absorption of approximately 0.3 is observed from 1150 Hz to the end of the tested frequency range. Incorporating a cavity into the acoustic system increases sound absorption: the maximum value of the sound absorption coefficient increases and the range of increased sound absorption widens. For an acoustic system with a composite sound-absorbing plate and a cavity, the peak indicating the resonant frequency becomes higher and higher and increasingly shifted towards lower frequencies as the length of the cavity increases. Starting from a cavity length of 20 mm, this peak is very clear and indicates a maximum value of the sound absorption coefficient of 0.6, occurring at a frequency of 430 Hz. When increasing the length of the cavity, the maximum value of the sound absorption coefficient increases and reaches the highest possible value, i.e. 1.0 at a frequency of 270 Hz for a cavity length of 100 mm.

Based on theoretical formulas, the resonant sound frequency for different cavity length “d” was calculated and compared with the experimental results.

Resonant sound frequency for given cavity length is calculated below:

Q was calculated for $\:{\text{f}}_{\text{r}\text{e}\text{s}}=550\:\text{H}\text{z}$

$$\:Q=66.649\:Hz\bullet\:{m}^{-2}$$

Sample calculations of theoretical resonant sound frequency:

for $$\:d=1\:cm=0.01\:m$$

$$\:{\text{f}}_{\text{r}\text{e}\text{s}}=666.49\:\text{H}\text{z}$$

**Fig. 7:**
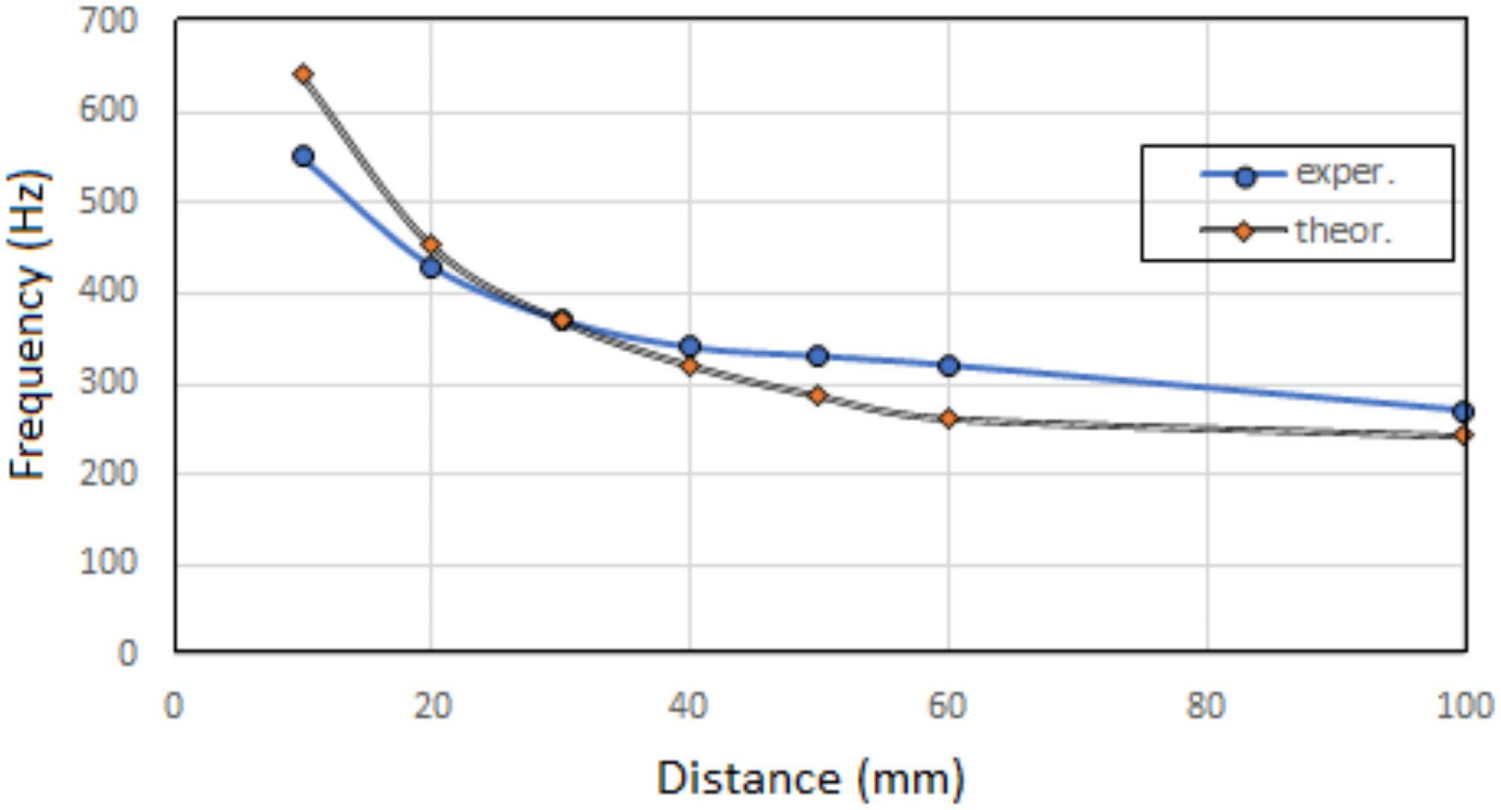
Resonant sound frequency – cavity length dependence for acoustic system with absorbing plate containing a polymer surface layer, situated perpendicularly to the direction of the sound wave.

The agreement between theoretical and experimental results of resonant sound frequency for given cavity length is confirmed by the curves in Fig. 7.

**Fig. 8:**
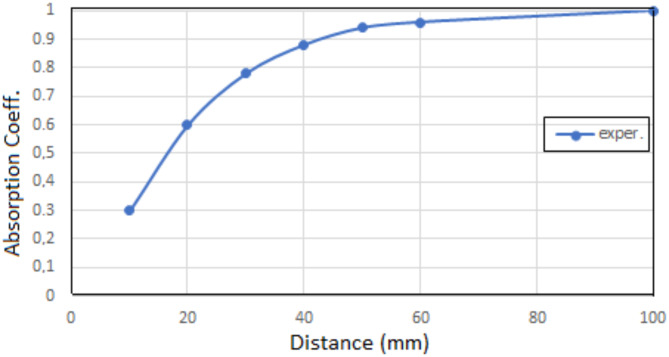
Sound absorption – cavity length dependence for acoustic system with absorbing plate containing a polymer surface layer, situated perpendicularly to the direction of the sound wave.

As the length of the cavity increases, the range of maximum absorption occurring at the resonant frequency shifts towards lower frequencies, Fig. 7, and the maximum value of the sound absorption coefficient increases, Fig. 8.

#### Acoustic system with absorbing plate situated at arctg2 to the direction of the sound wave - variant b

The scheme of the system is shown in Fig. 1b Variant b. The dependence of the sound absorption coefficient of the acoustic system on the length of the cavity was investigated. In the tests, a cavity in the form of a cylinder obliquely cut on one side was used, the front of which was an absorbing plate containing a polymer surface layer, situated at arctg2 to the direction of the sound wave. The shortest distance “d”, called cavity length, between the absorbing plate and the opposite back wall was up to 50 mm.

**Fig. 9:**
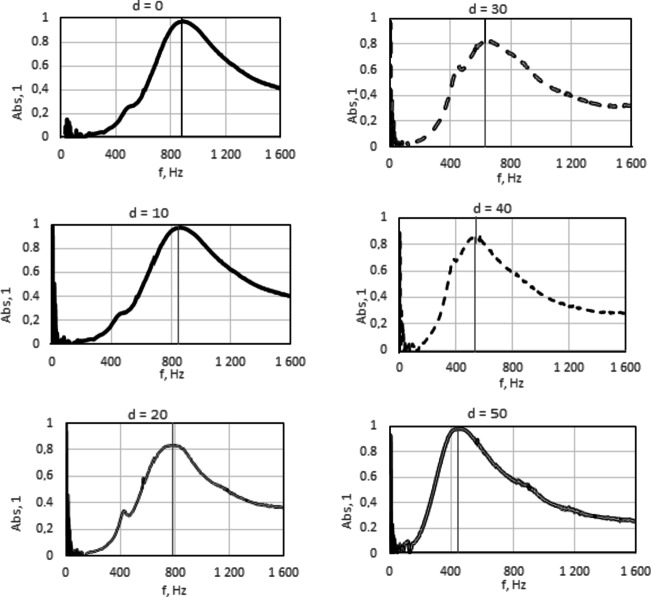
Sound absorption of the acoustic system with an absorbing plate containing a polymer surface layer, situated at the angle arctg2 to the direction of the sound wave - different cavity length (separate drawings for subsequent cavity length “d”).

In order to facilitate the analysis of the dependence of the sound absorption coefficient on the sound frequency for an acoustic system with the absorbing plate situated at an angle arctg2 to the direction of the sound wave and a different cavity length, individual plots are shown in Fig. 9 separately. As in the case of the perpendicular position of the absorbing plate to the direction of the sound wave, increasing the distance between the absorbing plate situated at the angle arctg2 to the direction of the sound wave causes a shift of the maximum sound absorption towards lower sound frequencies.

In an acoustic system with a cavity, the location of the composite absorbing plate, consisting of a fibrous layer and a polymer surface layer, at an angle arctg2 to the incident sound wave, causes that the ranges of the highest absorption refer to sounds of slightly higher frequencies than in the case of perpendicular location of the absorbing plate. Moreover, the right arm of the peak is wider open, so the high absorption frequency range is wider. This is confirmed by the exemplary graphs of the dependence of the sound absorption coefficient on the sound frequency for acoustic systems in Figs. 6 and 9.

The extension of the high absorption frequency range of in the case of an oblique location of the absorbing plate in relation to the direction of the incident sound wave results from the fact that the length of the cavity in different places of its cross-section is different, which results from the angle between the absorbing plate and the direction of the incident sound wave. The cavity can be considered as a set of elementary cavities of different lengths. Each elementary cavity is characterized by its own resonant frequency as shown in the previous section “Acoustic system with absorbing plate situated perpendicularly to the direction of the sound wave - variant a” and therefore the resonant frequency range is expanded.

As the cavity length increases, the high absorption range shifts towards the lower frequencies. This dependence is similar to the cavity with absorbing plate situated perpendicular to the direction of the sound wave.

#### Sound absorption of the acoustic system with absorbing plate and resonant cavity - the influence of the angle between the absorbing plate and the direction of the incident sound wave

**Fig. 10:**
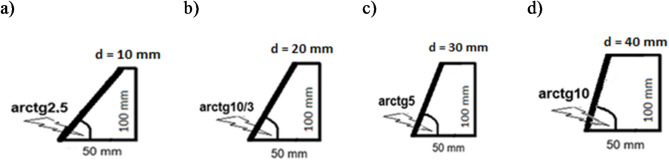
Acoustic system with an absorbing plate situated at different angle to the direction of the sound wave; (a) arctg2.5; (b) arctg10/3; (c) arctg5; (d) arctg10.

The dependence of the sound absorption coefficient of the system on the angle between the absorbing plate and the direction of the incident sound wave in the acoustic system was investigated. In the tests, a cavity was used in the form of a cylinder obliquely cut on one side, the front of which was an absorbing plate situated at a varied angle to the direction of the sound wave. The shortest dimension between the absorbing plate and the opposite back wall, “d”, was changed from 10 mm to 40 mm, and the longest dimension was 50 mm in each case, Fig. 1b Variant b and Fig. 10.

By changing the angle between the absorbing plate and the direction of the incident sound wave in the acoustic system, it is possible to control the sound absorption in the low frequency range. The greater the angle, the maximum absorption is noted for lower sound frequencies.

**Fig. 11:**
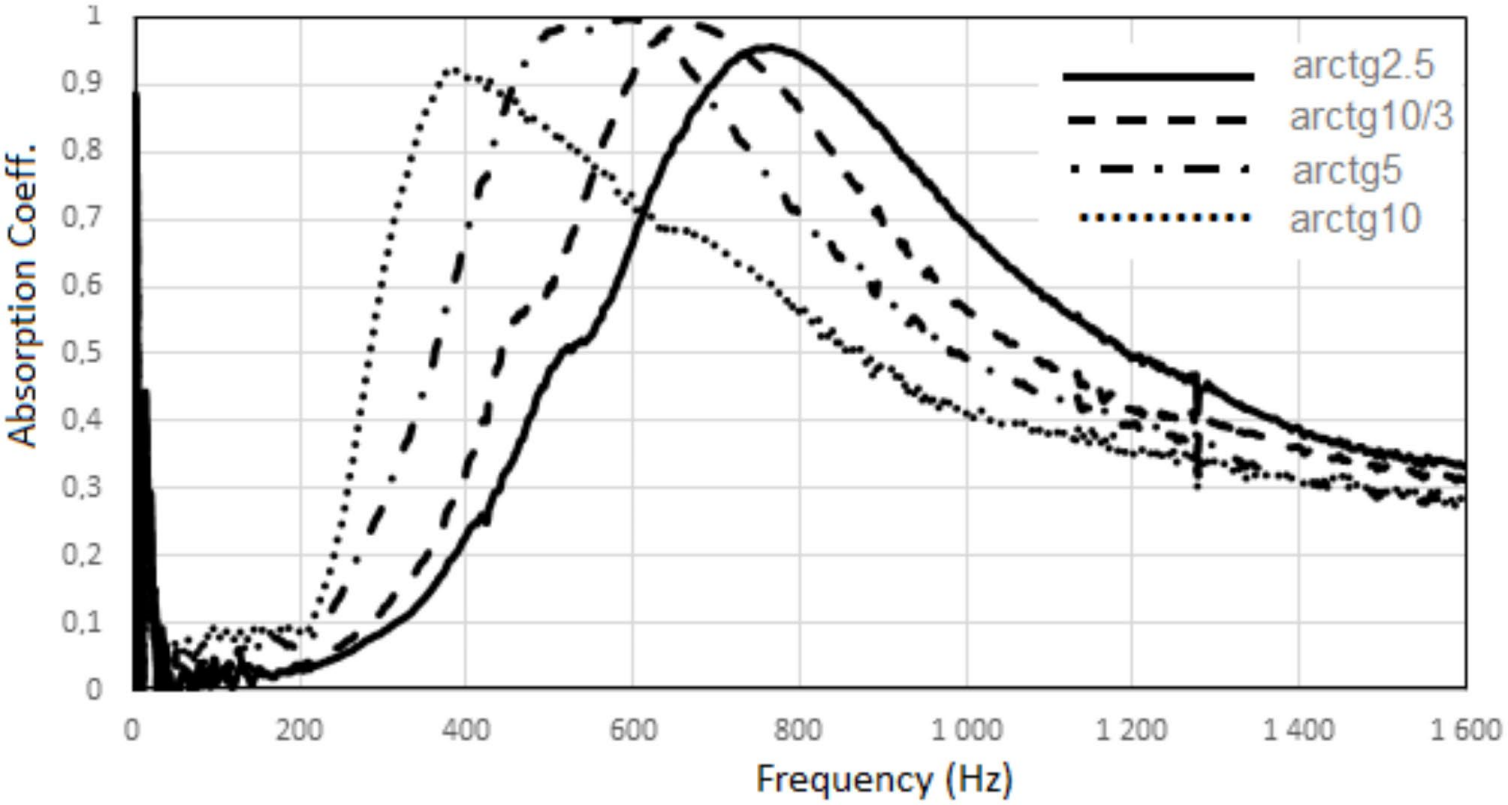
Sound absorption of the acoustic system – different angle between the absorbing plate containing a polymer surface layer and the direction of the incident sound wave.

For example, for a system with a plate containing the polymer surface layer the change of the angle from arctg2.5 to arctg10 causes the maximum absorption (0.9-1.0) to shift from the frequency of approx. 745 Hz to approx. 375 Hz, Fig. 11.

The obtained results show that by changing the angle between the absorbing plate containing a polymer surface layer and the direction of the incident sound wave in the acoustic system, it is possible to influence the frequency range of sounds most absorbed by the material. With this method, it is possible to effectively ensure high absorption of low-frequency sounds.

**Fig. 12:**
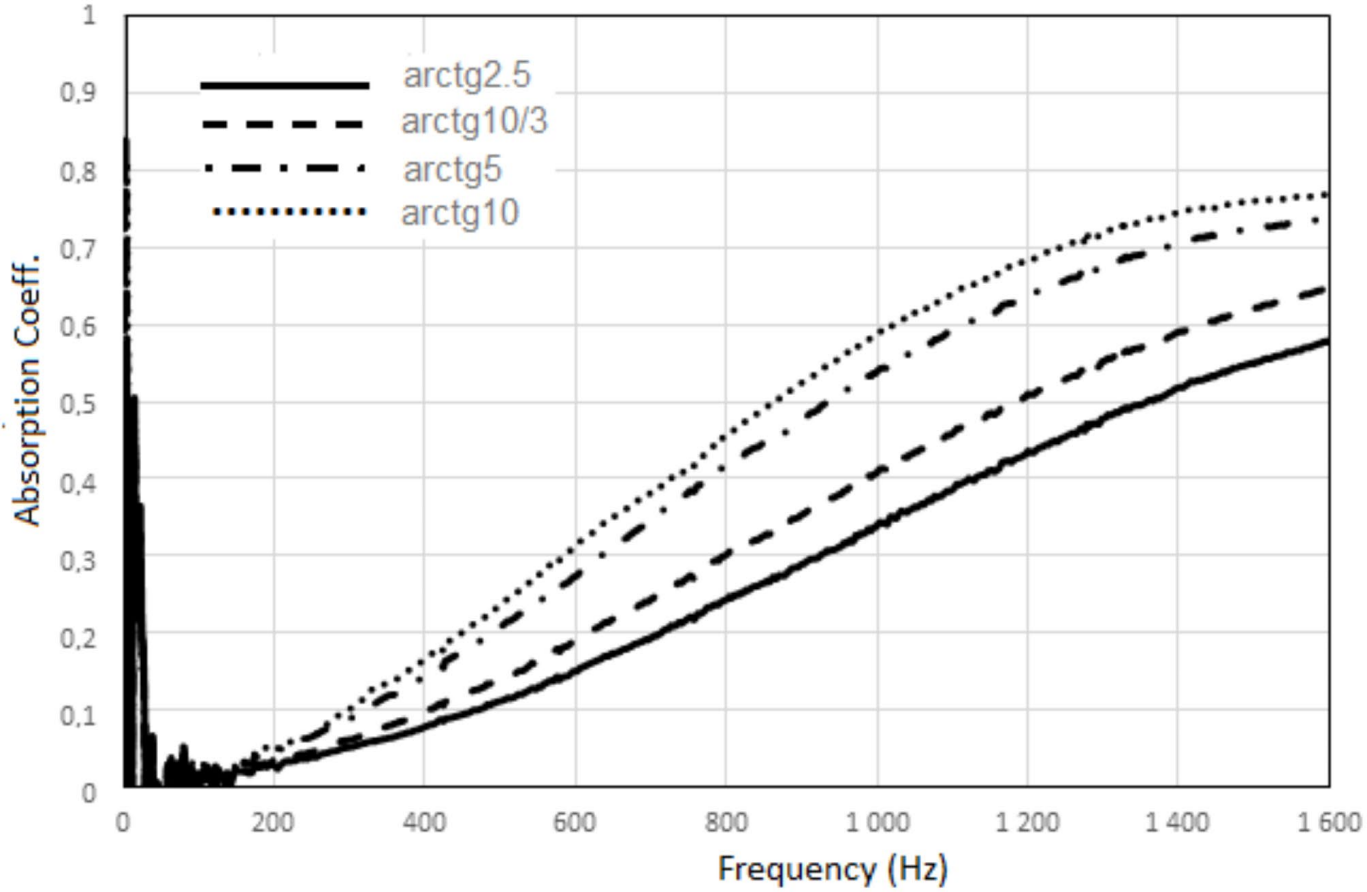
Sound absorption of the acoustic system – different angle between the reference absorbing plate without a polymer surface layer and the direction of the incident sound wave.

For comparison, a system with an absorbing plate without a polymer surface layer has no visible resonant sound frequency and the maximum sound absorption for the entire tested frequency range is lower (0.58–0.76) and occurs at higher frequencies (1600 Hz) than in the case of a system with an absorbing plate with a polymer surface layer. Generally, the greater angle between the absorbing plate and the direction of the incident sound wave in the acoustic system, the greater sound absorption, Fig. 12.

## Conclusions

Achieving high absorption of low-frequency sounds, i.e. up to 1600 Hz, is possible thanks to the use of an acoustic system consisting of an absorbing plate and an air cavity. The absorbing plate, the main part of which is a fibrous composite layer, should also include a polymer surface layer on the side of the incident sound wave.

Generally, this design of the absorbing plate promotes maximum sound absorption at lower frequencies and at a higher level than in the case of a fibrous composite plate without a polymer layer.

For a system with an absorbing plate containing a polymer surface layer and an air cavity, a resonant frequency is observed. For such a system, when the absorbing plate is placed perpendicular to the direction of the sound wave, as the length of the cavity increases, the range of maximum absorption, occurring at the resonant frequency, shifts towards lower frequencies, and the maximum value of the sound absorption coefficient increases.

In the case of an oblique position of the absorbing plate in relation to the direction of the incident sound wave, an extension of the high-absorption sound frequency range is observed. As the cavity length increases, the maximum absorption, which occurs at the resonant frequency shifts towards lower frequencies.

By changing the angle between the absorbing plate with a polymer surface layer and the direction of the incident sound wave in the acoustic system, it is possible to change the frequency range of the sounds that are maximally absorbed. There is a relationship that the larger the angle, the maximum absorption is observed for sounds with lower frequencies. Increasing the length of the cavity in the tested range, i.e. up to 50 mm, with the angle between the absorbing plate and the direction of the incident sound wave in the acoustic system equal to arctg2, shifts the maximum absorption towards lower and lower sound frequencies. By positioning the absorption plate in the acoustic system at an angle to the direction of the incident sound wave, it is possible to expand the frequency range of strongly absorbed sounds.

## Data Availability

The datasets used and/or analysed during the current study available from the corresponding author on reasonable request.
